# Dosimetric comparison between intensity-modulated radiation therapy and volumetric-modulated arc therapy to enhance bladder and bowel

**DOI:** 10.25122/jml-2022-0317

**Published:** 2023-09

**Authors:** May Zeki Saddik, Fatihea Fatihalla Hassan

**Affiliations:** 1 Department of Pharmacology/ Medical Physics/ and Clinical Biochemistry, College of Medicine, Hawler Medical University, Erbil, Iraq

**Keywords:** Dosimetric, intensity modulated radiation therapy, volumetric modulated arc therapy, prostate cancer, rectum sparing

## Abstract

Prostate cancer is the second most common cancer in men. Two common radiotherapy techniques, intensity-modulated radiation therapy (IMRT) and volumetric-modulated arc radiotherapy (VMAT), are used for treatment. This study aimed to compare the two techniques for sparing the bladder and bowel. Computed tomography data from prostate cancer patients were analyzed to define the clinical target volume (CTV) and planning target volume (PTV). Treatment plans were generated with Monte Carlo algorithms, and dosimetric analysis was performed using the Monaco Treatment Planning System (TPS). We compared IMRT and VMAT for prostate cancer PTV coverage (% Ref. Volume), with VMAT showing slightly better coverage (98.885±1.704) compared to IMRT (98.594±0.923). VMAT also demonstrated improved PTV conformity. Additionally, VMAT was superior in sparing the bladder (% V4500<40%), while IMRT performed better in bowel preservation (mean Ref. volume CC<195).

## INTRODUCTION

Cancer, a leading cause of mortality in developed nations, is predicted to increase in less developed countries as the population ages [1]. Prostate cancer is the 2^nd^ most frequent cancer in males and the 6^th^ most prevalent reason for cancer mortality. 1,276,000 cases and 359,000 deaths due to prostate cancer were identified in 2018 [2]. Prostate cancer is predominantly prevalent among elderly males, as more than 75% of cases are detected in men over the age of 65 [3]. According to studies, about three-quarters of prostate cancers occur in developed countries [2]. Starting from the 1970s, there has been a notable surge in the occurrence of prostate cancer in several Asian nations, including Singapore, China, and Japan. Consequently, it is experiencing an upward trend globally [4]. Advanced age is the leading risk factor for prostate cancer, being more common in black men and those with first-degree relatives with prostate cancer [3]. Approximately 30% of patients with prostate cancer relapse after definitive treatment [5]. Currently, treatments used for prostate cancer include taxane-based chemotherapeutic agents, prostatectomy, and radiotherapy [6]. Because invasive treatments can cause complications such as bleeding and damage to other tissues during surgery, the use of new and safe methods to treat prostate cancer is recommended [1]. When compared to other therapies, radiation therapy (RT) is one of the safest options for treating prostate cancer [7]. Most non-metastatic prostate cancer patients have a survival rate of over 10 years, so it is important to choose RT techniques with minimal toxicity [8]. Higher doses of radiotherapy have been reported to control 15 to 20 percent of prostate cancer [9]. However, some of the toxic effects of radiation can damage parts of the gastrointestinal tract, including the bladder and bowel [10].

Today, two types of RT are used to treat cancer, including intensity-modulated radiation therapy (IMRT) and volumetric-modulated arc radiotherapy (VMAT) [11]. IMRT accurately distributes beams by optimizing computer-determined non-uniform beam intensities and has emerged as an advanced technique [12]. IMRT can also focus a relatively large dose of radiation on the cancerous area while minimally damaging adjacent noncancerous tissues [13]. VMAT can also provide a very coherent dose distribution in a short period, which is why it has attracted the attention of the RT community [14]. In IMRT, fewer than 10 specific beam angles are typically created, whereas VMAT involves a substantial variety of beam directions that deliver doses in an arc during gate rotation [15]. Although more than one arc is sometimes used in VMAT, the beam modulation level is much lower than each beam in a fixed field IMRT [16, 17]. The VMAT approach can cut the beam illumination duration by up to 55% while providing IMRT-quality dosimetry [9]. While VMAT has demonstrated enhanced delivery efficiency compared to IMRT, it remains uncertain whether VMAT offers superior quality for the treatment of prostate cancer [18, 19].

This study aimed to compare IMRT and VMAT techniques for prostate cancer treatment, focusing on their effectiveness in achieving optimal target coverage while minimizing the impact on critical structures like the bladder and bowel, which is assessed through evaluating clinical and dosimetric outcomes.

## MATERIAL AND METHODS

### Study design and participants

This study involved ten patients diagnosed with prostate cancer, and each patient underwent two separate radiation therapy (RT) techniques: intensity-modulated radiation therapy and volumetric-modulated arc radiotherapy. Subsequently, a total of 20 treatment plans were generated for all ten patients. The prescribed radiation dose for each treatment plan was 6000 cGy. The research, conducted in 2022, utilized the Electa Synergy linear accelerator (LINAC) at the Zhinawa Cancer Center. The LINAC incorporates three-photon energy levels (6, 10, and 18 MV) and eight electron energy levels (4, 6, 8, 10, 12, 15, 18, and 22). Additionally, the LINAC is equipped with a Multi-Leaf Collimator, making it suitable for precise radiation therapy.

### Optima CT scanner

**Figure 1:**
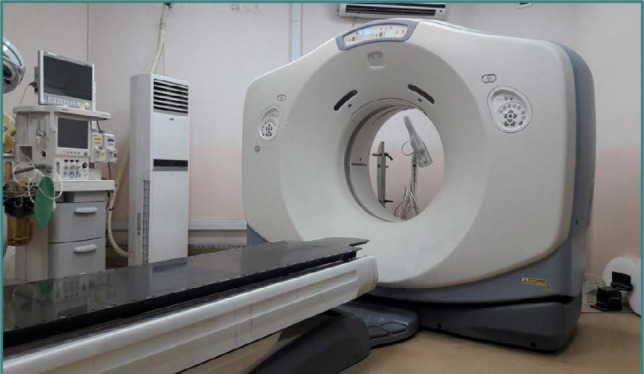
Optima CT scanner

The Optima CT 580 RT scanner from General Electric Healthcare-USA was employed to acquire patient images. This 80cm big bore CT scanner is designed explicitly for radiotherapy and includes a flat RT couch. The Optima 580 is a 16-slice scanner, meaning it captures 16 slices in a single rotation of its gantry and offers various slice thickness options, ranging from 0.625mm to 10mm. Moreover, this scanner can acquire 4D-CT images, accommodating helical and axial scanning techniques (Figure 1).

### Monaco planning system

The radiotherapy Treatment Planning System (TPS) utilized in this research was Monaco, developed by Elekta. Monaco is a highly versatile software capable of planning various radiotherapy techniques, such as 3D, IMRT, VMAT, SRS, and Brachytherapy, with exceptional precision. It achieves this high level of accuracy through the utilization of the Monte Carlo algorithm, recognized as the most precise dose calculation method currently available.

Monaco TPS version 5.00.02 is available at the Zhinawa Cancer Center (ZCC). It operates within a network environment consisting of three central high-capacity computer systems. These computers are equipped with Quad-core Intel Xeon processors running at 2.93GHz, 24GB of DDR3 RAM, and a storage capacity of 4 TB. Additionally, the Monaco TPS is seamlessly connected to the central network of the facility.

This research formulated VMAT and IMRT treatment blueprints employing a 10 MV photon beam through the clinical Monaco Version 5.11.01 Treatment Planning System. VMAT schemes involving a pair of arcs were computed for all participants in this study using the Monte Carlo computational approach.

### IMRT and VMAT

Several oscillating radiation intensities in IMRT radiotherapy, a cutting-edge technique for administering three-dimensional therapy, deliver the highest intended dosage to tumor tissue while causing the least amount of undesired radiation to reach healthy tissues. This method is achieved by subdividing each radiation beam into smaller beams and modulating the intensity of each beam. Advanced computer technology has facilitated software development for IMRT treatment design and delivery. As a result, it delivers a consistent dose to tumors and vital organs while protecting them from radiation dose by dropping the dose slope in healthy tissues. IMRT is also an advanced method of radiotherapy used to treat cancerous and non-cancerous tumors. It employs sophisticated technology to adjust photon and proton beams based on the tumor's specific form. IMRT employs numerous tiny photons and protons with varying strengths to precisely target and irradiate the tumor.

**Figure 2:**
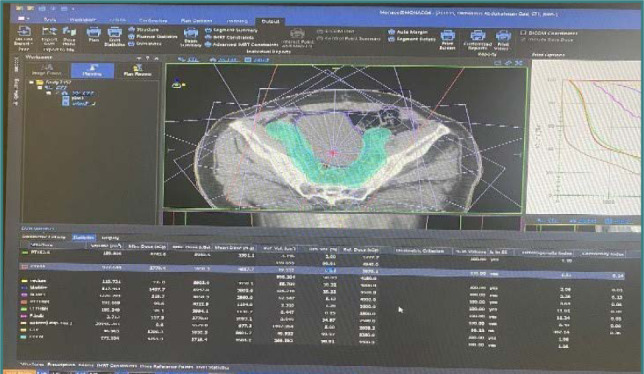
IMRT prostate treatment planning for a representative patient

The intensity of each beam is managed, and the configuration of the beam undergoes alterations throughout each treatment session. The fundamental goal of IMRT is to reach the prescribed radiation dose within the target area while safeguarding the integrity of adjacent healthy tissue and minimizing the adverse consequences of the therapy. Additionally, IMRT can create dose distributions that are not uniform, allowing for the simultaneous delivery of distinct doses to distinct regions within the target volume during each treatment fraction. IMRT is founded on a “reverse treatment design” strategy that employs non-uniform radiation patterns to optimize the dose distribution within the target tissue. In IMRT, employing numerous fixed-angle radiation beams is often necessary, a practice that extends the duration of treatment delivery as shown in Figure 2. This extension may have adverse implications for patient comfort during treatment, the repeatability of treatment positioning, and the possibility of movement between treatment sessions. Furthermore, prolonging treatment times raises concerns regarding the radiobiological impact, as it allows for increased cell repair and proliferation among cancerous cells during the extended therapy duration.

**Figure 3:**
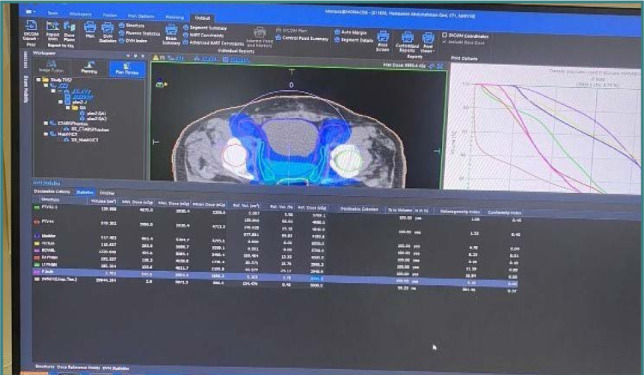
VMAT prostate treatment planning for a representative patient

**Figure 4:**
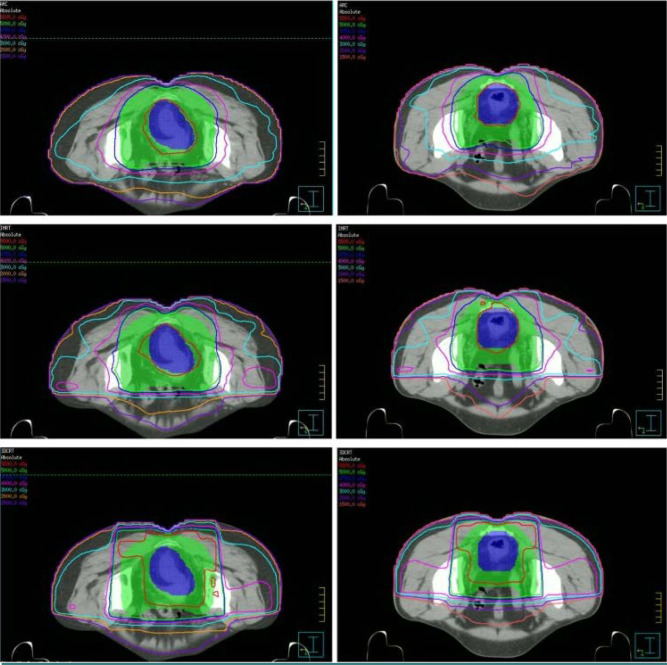
Dose distributions of three planning techniques, 3DCRT, IMRT, and VMAT

One form of IMRT is VMAT radiation. This method, utilizing one or sometimes multiple radiation beams, focuses the radiation precisely on the tumor, resulting in a reduced treatment duration as illustrated in Figure 3. In this approach, the machine rotates around the patient, a maneuver designed to minimize radiation exposure to adjacent organs, thereby contributing to the reduction of potential adverse effects for better dose distribution when compared with other techniques (Figure 4).

### Intervention

In the beginning, computed tomography data of prostate cancer patients were simulated, and treatment planning was performed in the TPS. CTV was selected from the studied tissues, and PTV and CTV were expanded by 5 mm in all directions.

**Figure 5:**
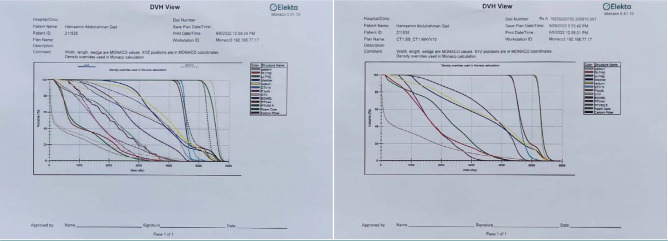
Dose-volume histograms of the PTV (prostate) in two treatment planning techniques

Nine fields produced IMRT beams with gate angles of 0, 50, 100, 150, 205, 255, and 310. Then, VMAT beams were generated using two complete arcs (one clockwise and one counterclockwise). Ultimately, the treatment plan underwent Monte Carlo algorithm execution, and a dosimetric examination ensued. Parameters, including the minimum dose, maximum dose, mean dose, and coverage of the Planning Target Volume (PTV), were computed using a dose volume histogram (DVH) (Figure 5). Additionally, the conformity index (CI) was appraised using the formula provided below.


$$Conformity\;Index=\frac{Volume\;of\;prescription\;dose}{Volume\;of\;the\;PTV}$$

The value D2% signifies the highest dose that will be administered to 2% of the PTV, while D98% corresponds to the lowest dose calculated for 98% of the PTV. The assessment of dose homogeneity, denoted as the homogeneity index (HI), was determined using the formula outlined in the 83rd International Association of Radiation Units (ICRU) report for the target tissue, as indicated by the following equation.


HI=D2%⋅D98% Dp

The data for PTVs included acquiring D2% and D98% values via DVH analysis. D2% signifies the highest dose administered to 2% of the PTV, while Dp denotes the prescribed dose for the PTV. D98% represents the minimum dose calculated for 98% of the PTV.

### Data analysis

All the metrics were computed based on the information extracted from the DVH. Statistical assessments were conducted using IBM SPSS Statistics version 23 (SPSS Inc., Armonk, NY, USA). We utilized an independent Student's t-test to assess the differences between the two approaches, considering statistical significance as a p-value less than 0.05.

## RESULTS

This study evaluated the dosimetric outcomes of prostate cancer treatment using IMRT and VMAT techniques in ten patients. Each patient underwent treatment with both modalities, resulting in 20 treatment plans. The prescribed dose for all plans was 6000 cGy. Table 1 shows results, including volume cc, mean dose Gy, Ref. Volume Percent, Ref. dose Gy, Ref. Volume CC, HI, CI, D2%, and D98%.

**Table 1:** PTV cover parameters comparison between IMRT and VMAT techniques

PTV	Mean ± SD		p (independent student t-test)
	IMRT	VMAT	
Volume CC	507.136±499.603	507.136±499.603	1
Mean dose Gy	5628.041±1719.855	5379.29±871.621	0.6
Ref. volume. %	98.594±0.923	98.885±1.704	0.05
Ref. dose	5275.3±386.272	5275±386.272	1
Ref. volume CC	26.371±28.474	78.948±75.565	0.05
CI	1.197±0.078	1.143±0.054	0.04
HI	0.075±0.049	0.437±0.008	0.3
Maximum dose (D2%)	6471.12±814.457	6324.31±742.341	0.357
Minimum dose (D98%)	4500	4500	1

CI: Conformity index, HI: Homogeneity index, PTV: Planning target volume, VMAT: Volumetric-modulated arc therapy, IMRT: Intensity-modulated radiotherapy, SD: Standard deviation

The mean CI in IMRT (1.197±0.78) was significantly higher than the mean CI in VMAT (1.143±0.054) (p≤0.04), indicating that IMRT provides superior conformity in target coverage compared to VMAT. However, there was no significant difference in the mean HI (0.075±0.049) in IMRT compared to the mean HI (0.437±0.008) in IMRT (p≤0.3).

Regarding PTV coverage for prostate cancer, the Ref. Volume. % was 98.594±0.923 for IMRT and 98.885±1.704 for VMAT. VMAT demonstrated a slightly higher Ref. Volume. %, suggesting better coverage compared to IMRT.

The findings indicated no significant disparity in the mean dose in Gy, D2%, or the mean D98% between the two diagnostic modalities, IMRT and VMAT (Table 1).

The mean Ref. volume % V4500<40% (indicating the volume receiving 4500 cGy should be less than 40%) was 31.184±12.051 in IMRT and 0.052±0.104 in VMAT. The lower value in VMAT indicates its superiority in sparing the bladder in terms of % V4500<40%. Similarly, the mean Ref. volume % V6000<5% were evaluated for bladder sparing, with values of 4.026±1.120 for IMRT and 10.95±9.567 for VMAT, respectively. The lower value in IMRT suggests that IMRT is the superior technique for sparing the bladder in terms of % V6000<5% (Table 2). For the bladder, the constraint was V6000<5%, or the percentage volume of the bladder receiving 6000 cGy should be less than 5%.

**Table 2:** Comparison of Ref. volume. % (V4500<40%) and Ref. volume. % (V6000<5%) for bladder

	Mean ± SD		p (independent student t-test)
	IMRT	VMAT	
Ref. volume. % V4500<40%	31.184±12.051	0.052±0.104	0.001
Ref. volume. % V6000<5%	4.026±1.120	10.95±9.567	0.003

PTV: Planning target volume, VMAT: Volumetric-modulated arc therapy, IMRT: Intensity-modulated radiotherapy, SD: Standard deviation

The mean Ref. volume CC for the bowel, with a target value of less than 195 cc, was evaluated in both IMRT and VMAT techniques. In the IMRT group, the mean value was 26.371±28.371 cc, while in the VMAT group, it was significantly higher at 78.948±75.565 cc. These results indicate that IMRT and VMAT achieved values below the 195-cc threshold. However, IMRT demonstrated superior bowel sparing compared to VMAT, with significantly lower mean values (Table 3).

Figure 5 presents dose-volume histograms (DVHs) for all patients treated with IMRT and VMAT techniques. The results reveal a favorable trend in HI for VMAT, indicating its potential for effective disease diagnosis and control. Additionally, the CI also demonstrated a superior trend in the VMAT method compared to IMRT.

**Table 3:** Comparison of dose parameters between IMRT and VMAT techniques for bowel

	Mean ± SD		p (independent student t-test)
	IMRT	VMAT	
Ref. volume CC <195 cc	26.371±28.371	78.948±75.565	0.005

VMAT: Volumetric-modulated arc therapy, IMRT: Intensity-modulated radiotherapy, SD: Standard deviation

## DISCUSSION

Our comparison between IMRT and VMAT techniques for treating prostate cancer PTV revealed several key findings. The evaluation of Ref. Volume. % values clearly indicate that VMAT was better than IMRT in providing better overall coverage, as higher values were observed in VMAT. The higher Ref. volume% in VMAT plans indicates superior PTV coverage compared to IMRT. The VMAT technique was most effective in sparing the bladder when the constraint was V4500% of the bladder <40%, whereas IMRT performed better in sparing the bladder when the constraint was V4500% of the bladder <5%. Additionally, IMRT outperformed VMAT in minimizing the volume (cc) of the bowel when the constraint was (bowel volume cc<195 cc).

The effects and benefits of radiotherapy in prostate cancer have been demonstrated in several studies [20]. Prior research has similarly demonstrated that IMRT therapy outperforms alternative treatment modalities [21, 22]. Additionally, when comparing newer radiotherapy methods with the established IMRT technique, these innovations have only shown marginal improvements [23, 24]. Subsequently, in this study, IMRT was considered the preferred choice for bladder and bowel preservation compared to VMAT. Earlier research has indicated the complexity of determining the optimal treatment strategy and approach for prostate cancer [25]. Nonetheless, IMRT and VMAT presently enjoy extensive adoption in the realm of treatment, and endeavors persist to enhance the efficacy of these techniques [26, 27].

In Calmels *et al*. study [28], 60 patients with anal, rectal, and prostate cancer, 20 each, were examined retrospectively. The finding showed that the homogeneity index was lower, and the average dose was higher for some organs. It also showed better results for conformity and homogeneity indices in the IMRT method compared to the VMAT method, while in the present study, VMAT was shown to be the better method.

In this study, the PTV for the prostate was 98%, which is consistent with the target index of more than 95%. In the review conducted by SKD Majumdar *et al*. [29], the PTV and distance for organs were compared using three methods (3D-CRT, IMRT, and VMAT). The finding of this study showed that both IMRT and VMAT methods led to improved coverage for 95% of PTV. These outcomes align with the findings presented in the current research, which demonstrate enhancements in PTV coverage.

The results indicate that the VMAT plan performs better when considering the mean Ref. volume % V4500<40, suggesting its superiority over the IMRT plan. On the other hand, the IMRT plan performs better when examining Ref. volume % V6000<5%. These findings are consistent with a prior study by Shawata *et al*. [30], which also observed similar dose values for 4500 cGy and 6000 cGy, aligning with the target index values.

The homogeneity index is suitable for dose distribution in the target volume or tissue, but the factors affecting this index are not well identified. The homogeneity and adaptation indices were different in different studies according to the examined organ. While some tissues and organs may exhibit favorable indices, others may not [31]. In a study conducted by Afrin, the results indicated that the homogeneity index (HI) improved with the VMAT method compared to IMRT [32]. These findings are not consistent with the results of the present study, where the HI was better with the IMRT method.

The examination of the conformity index also revealed a significant difference between the two methods, IMRT and VMAT, with a relative improvement seen in the IMRT method. This finding contrasts with a study by Nguyen *et al*., which compared IMRT and VMAT therapies [33]. In their study, no significant variance was observed in the measures of homogeneity and conformity, in contrast to the outcomes observed in our current investigation. However, in another study, a statistically significant difference was observed in the homogeneity and compliance indices [34], which is consistent with the results of the present study.

In the current study, the analysis of additional dosimetric indices, such as mean dose and maximum dosage, did not reveal any differences between IMRT and VMAT procedures. This finding is comparable with the findings of earlier studies, which did not reveal any differences [35, 36].

Today, advanced radiotherapy technologies such as IMRT are increasingly widespread. However, the complexity of treatment design and delivery technologies, such as the use of multi-blade collimators and the importance of their correct location and modeling in the treatment design system, increase the number of display units due to the decrease in the average width of the narrow beam window, which increases the share of indirect beams and uncertainty in the total received dose. The use of posterior oblique beams in severely modified radiotherapy and the increased likelihood of attenuation of the radiating beam by the treatment bed have prevented patients from achieving the desired dose distribution in the treatment process [37, 38].

Therefore, to calculate the correct dose of RT and the optimal delivery of the treatment plan to the patient, a great effort has been made to diagnose the errors as accurately as possible using various quality assurance processes before treating patients.

In addition to the common quality assurance method of gamma index with different criteria and the use of electronic portal images during treatment, various methods involving artificial intelligence and neural networks have been studied for detecting errors that can impact the treatment process. These methods have been used to detect spatial errors in multi-blade collimators and linear accelerator settings [39]. It is obvious that software development and artificial intelligence, with improvements in design and time, advanced quality assurance, and increased security in reviewing treatment plans, will be an important step in identifying and classifying uncertainties in RT and its correct delivery in the future [40].

## LIMITATIONS

Because the information used in this study has a retrospective trend and is affected by possible biases caused by retrospective studies, it is recommended to use prospective studies in future studies. Also, considering the relative improvements observed in homogeneity and conformity indices, exploring additional dosimetric aspects in future investigations is advisable.

## CONCLUSION

In the present study, the effects of prostate radiotherapy were evaluated by two techniques: IMRT and VMAT. Both techniques were assessed using the CI and HI indexes, as well as the PTV ref volume% or PTV cover. The results indicated that the VMAT technique outperformed IMRT. VMAT technique was the best in sparing the bladder in % V4500<40 %. Also, the lower value of IMRT shows that the IMRT plan was better than the VMAT plan. IMRT technique was the best in sparing the bladder in % V600<5 %. The mean Ref. volume CC<195 showed that the IMRT technique was better for sparing the bowel. These results suggest that IMRT and VMAT are viable treatment options for this condition. Considering that the homogeneity and conformity indices in this study showed a relative improvement, it is recommended that more aspects of dosimetric indices be further investigated.

## Personal thanks

The authors express their gratitude to the entire team at the Department of Radiology, Erbil Teaching Hospital, Erbil, Iraq, for their collaborative efforts and for providing the necessary facilities to facilitate this research.
